# Long Term Norovirus Infection in a Patient with Severe Common Variable Immunodeficiency

**DOI:** 10.3390/v14081708

**Published:** 2022-08-02

**Authors:** Loa Ottosson, Marie Hagbom, Rikard Svernlöv, Sofia Nyström, Beatrice Carlsson, Mattias Öman, Magnus Ström, Lennart Svensson, Åsa Nilsdotter-Augustinsson, Johan Nordgren

**Affiliations:** 1 Division of Molecular Medicine and Virology, Department of Biomedical and Clinical Sciences, Linköping University, 58185 Linköping, Sweden; loa.ottosson@regionostergotland.se (L.O.); marie.hagbom@liu.se (M.H.); sofia.nystrom@regionostergotland.se (S.N.); beatrice.carlsson@gmail.com (B.C.); oman.mattias@gmail.com (M.Ö.); lennart.t.svensson@liu.se (L.S.); 2 Department of Gastroenterology and Hepatology, Linköping University, 58185 Linköping, Sweden; rikard.svernlov@regionostergotland.se (R.S.); magnus.f.strom@telia.com (M.S.); 3 Department of Clinical Immunology and Transfusion Medicine and Department of Biomedical and Clinical Sciences, Linköping University, 58185 Linköping, Sweden; 4 Division of Infectious Diseases, Department of Medicine, Karolinska Institute, 17111 Stockholm, Sweden; 5 Infectious Diseases/Division of Inflammation and Infection, Department of Biomedical and Clinical Sciences, Linköping University, 58185 Linköping, Sweden; asa.nilsdotter-augustinsson@liu.se

**Keywords:** norovirus, chronic, evolution, CVID, ribavirin

## Abstract

Norovirus is the most common cause of acute non-bacterial gastroenteritis. Immunocompromised patients can become chronically infected, with or without symptoms. In Europe, common variable immunodeficiency (CVID) is one of the most common inborn errors of immunity. A potentially severe complication is CVID-associated enteropathy, a disorder with similar histopathology to celiac disease. Studies suggest that chronic norovirus infection may be a contributor to CVID enteropathy, and that the antiviral drug ribavirin can be effective against norovirus. Here, a patient with CVID-like disease with combined B- and T-cell deficiency, had chronic norovirus infection and enteropathy. The patient was routinely administered subcutaneous and intravenous immunoglobulin replacement therapy (SCIg and IVIg). The patient was also administered ribavirin for ~7.5 months to clear the infection. Stool samples (collected 2013–2016) and archived paraffin embedded duodenal biopsies were screened for norovirus by qPCR, confirming a chronic infection. Norovirus genotyping was done in 25 stool samples. For evolutionary analysis, the capsid (VP1) and polymerase (RdRp) genes were sequenced in 10 and 12 stool samples, respectively, collected before, during, and after ribavirin treatment. Secretor phenotyping was done in saliva, and serum was analyzed for histo-blood group antigen (HBGA) blocking titers. The chronic norovirus strain formed a unique variant subcluster, with GII.4 Den Haag [P4] variant, circulating around 2009, as the most recent common ancestor. This corresponded to the documented debut of symptoms. The patient was a secretor and had HBGA blocking titers associated with protection in immunocompetent individuals. Several unique amino acid substitutions were detected in immunodominant epitopes of VP1. However, HBGA binding sites were conserved. Ribavirin failed in treating the infection and no clear association between ribavirin-levels and quantity of norovirus shedding was observed. In conclusion, long term infection with norovirus in a patient with severe CVID led to the evolution of a unique norovirus strain with amino acid substitutions in immunodominant epitopes, but conservation within HBGA binding pockets. Regularly administered SCIg, IVIg, and ~7.5-month ribavirin treatment failed to clear the infection.

## 1. Introduction

Norovirus is the most common cause of acute, non-bacterial gastroenteritis worldwide, resulting each year in 685 million cases and 50,000 deaths amongst children [1]. The norovirus genus has a high genetic diversity and is divided into 10 genogroups based on amino acid sequence diversity in the major structural protein of the capsid (VP1). Genogroup II is predominant in humans and can be phylogenetically further separated into 26 capsid genotypes [2]. It can also be separated into 37 P-types based on nucleotide diversity in coding sequence of the RNA-dependent RNA polymerase (RdRp) [2]. Genotype GII.4 has been associated with more severe disease [3,4,5,6] and can be further divided into variants based on phylogenetic clustering, with new variants being recognized after becoming epidemic in at least two geographical locations [2].

The VP1 of GII.4 variants is structurally divided into shell (S; residues 1–215) and protruding (P) domains, and P is further divided into P1 (residues 216–280 and 416–540) and P2 (residues 281–415) [7]. While VP1 of human norovirus has the same intrinsic structure, the specific residue positions described here correspond to the GII.4 genotypes. The P2 domain constitutes the most surface-exposed part of the virus particle and contains both the histo-blood group antigen (HBGA) binding pockets, as well as immunodominant neutralizing antibody epitopes [8]. By determining monoclonal antibody and sera reactivity to norovirus virus-like particles (VLPs), epitope mapping using VLPs, and evolutionary analysis of emerging GII.4 variants, several immunologically important epitopes have been proposed: A (294–298, 368, 372–373), B (333, 389), C (339–341, 375–378), D (393–397), E (407, 411–414), and G (352, 355–357, 359, 364) [7]. Substitutions in at least some of these are described to correlate with the emergence of new epidemiologically important GII.4 variants [7,9,10,11]. Due to the difficulty in cultivating human noroviruses, an in vitro blocking assay using antibody blockade of VLP binding to carbohydrate ligands is used as a surrogate neutralization assay [10]. The A epitope is hypervariable and immunodominant and together with D it faces the most exterior part of the virus particle. Although a GII.4 virion contains the same number of A, E, and G epitopes, approximately 40% of serum antibody blockade responses target epitope A. Substitutions in this epitope correlate well with the emergence of new epidemiological significant strains [7,10,12,13,14].

The HBGA binding pockets of VP1 are highly conserved and believed to mediate norovirus infection through interactions with host HBGAs on the surface of the small intestinal mucosa. The proposed residues of the HBGA binding pockets include 343–345, 347, and 374, as well as 390–393 and 442–444 [15,16,17]. GII.4 norovirus has a clear host genetic susceptibility profile based on host HBGA expression, predominantly infecting so called secretors (~75–80% of the European population) that have α1,2 fucosylated HBGAs in secretions and on intestinal epithelia [18].

The RdRp is the major enzyme responsible for norovirus RNA replication and consequently a target for antiviral treatments [19]. Ribavirin, a nucleoside analogue, has been tested both in vitro [20,21] and in vivo [22,23,24] as a potential therapeutic agent against norovirus infection. The antiviral mechanism of ribavirin is still not fully understood, but mutagenesis and/or interference with RNA synthesis in the viral RdRp, as well as depletion of GTP levels in host cells are some proposed mechanisms of action [24,25].

In healthy immunocompetent individuals, norovirus infections are usually cleared within 21 days, with symptoms lasting 2–4 days [26]. However, prolonged and chronic norovirus infection can occur in individuals with primary or secondary immunodeficiency [27]. Symptoms in these individuals vary from prolonged diarrhea to asymptomatic shedding, with or without vomiting [28,29].

Common variable immunodeficiency (CVID) is one of the most common inborn errors of immunity in Europeans, with a prevalence of ~1:25,000. CVID is a heterogeneous condition characterized by increased susceptibility to infections and low levels of immunoglobulins due to dysfunctional B cells [27,30,31]. Many patients with CVID have a combined immunodeficiency with affected T cells as well [30].

Gastrointestinal problems are common in CVID, and diarrhea is commonly reported. Furthermore, approximately 5% of patients with CVID will be diagnosed with CVID-associated enteropathy [27,31], presenting with diarrhea, malabsorption, and histological findings similar to celiac disease, including duodenal villous atrophy and lymphocytic infiltration of the mucosa. The underlying mechanisms of CVID-related enteropathy is unknown. However, the subgroup of CVID patients that also has T cell dysfunction are predisposed to present with enteropathy [27,30,32].

In some cases, CVID-related enteropathy has been associated with chronic norovirus infection [27]. Chronic norovirus infections lasting for years have been observed in CVID patients, and upon viral clearance, these patients have experienced clinical and histological recovery, suggesting a causal connection [22,27]. These findings suggest that chronic norovirus infection may be a predominant cause of the enteropathy seen in CVID patients [27]. Oral immunoglobulins and nitazoxanide have been used to treat norovirus infections associated with immunosuppression [24,32]. A recent study successfully treated a norovirus infected CVID patient suffering from diarrhea with immunoglobulins administered naso-duodenally [24]. Furthermore, ribavirin has been linked to some instances of successful viral clearance in noroviral enteropathy associated with CVID [22].

Several studies have investigated norovirus intra-host evolution during chronic infections, mostly in transplant recipients receiving immunosuppressant drugs [15,28,33,34]. The evolutionary patterns of the VP1 gene in these studies have shown similarities to what is observed in norovirus infections in immunocompetent individuals, with amino acid substitutions in known antigenic epitopes.

In this study, we analyzed the evolution of norovirus VP1 and RdRp genes in a patient with CVID disease with chronic norovirus infection before, during, and after ribavirin treatment. The results are discussed in light of norovirus evolution and treatment strategies.

## 2. Materials and Methods

### 2.1. Patient Description

The study was approved by the regional ethical board of Linköping University (Dnr 2016/189-31). Informed written consent was obtained from the patient.

The patient had a severe form of CVID with dysregulation of T cells and a progressive loss of B lymphocytes. Among other effects, this led to panhypogammaglobulinemia, a profound decrease in all the major classes of immunoglobulins. Therefore, the patient received subcutaneous immunoglobulin replacement therapy (SCIg) at regular intervals. In 2009, the patient developed chronic gastrointestinal disease with recurrent symptoms of diarrhea, malabsorption, and weight loss. Initially, celiac disease was suspected based on histological findings in the duodenum. The patient’s condition did not improve on gluten-free diet. In 2013, the first norovirus PCR analysis was carried out on a fecal sample and showed infection with norovirus GII. Gastrointestinal tract histology from 2013 was consistent with an ongoing norovirus infection. Retrospective testing using qPCR of paraffin-embedded sections of duodenal samples from 2010 and 2011, collected after the development of the gastrointestinal disease, showed that the patient was already positive for norovirus GII in 2010 (Ct value 32.5).

Even though administered with adequate doses of SCIg, the patient continually showed suboptimal serum levels of immunoglobulins. Initially, increased doses of SCIg were evaluated, but because of failure to achieve an adequate serum level of immunoglobulins, intravenous immunoglobulin replacement therapy (IVIg) was introduced in 2015. IVIg was subsequently administered every third week along with SCIg. A correlation was seen between increased gastrointestinal symptoms and reduced serum immunoglobulin levels, possibly attributable to leakage and/or consumption of immunoglobulins in the chronically infected small intestine.

To attempt clearance of the confirmed norovirus infection, ribavirin treatment was initiated at 400 mg twice a day orally. The treatment continued for ~7.5 months in 2015. Serum concentrations of ribavirin were recorded once a week, except for when the patient was ill for other reasons than the norovirus infection, requiring admission to hospital. Target serum levels were set at 1000 ng/mL [22]. Serum levels varied in the range 561–2073 ng/mL. A weekly stool sample was also collected from the patient to be analyzed for norovirus and stored at −80 °C for later analyses (Table 1).

### 2.2. Blocking of Human GII.4 VLPs Binding to Pig Gastric Mucin

The patient’s secretor status was determined using *Ulex europaeus* agglutinin (UEA-I, Sigma Aldrich, Stockholm, Sweden), which recognizes Fucα1-2Gal-R present in the saliva of secretors, essentially as previously described [35]. Blocking-antibody titers against GII.4 norovirus VLPs were determined in one serum sample as previously described [33]. The blocking titers (BT_50_) were defined as the serum dilution at which the OD_450_ value was 50% of the OD_450_ value for the positive control (only VLP). A BT_50_ value of ≥160 was considered protective against norovirus gastroenteritis [34]. The blocking titers were determined using the Grimsby (isolated in 1995 [AJ004864]) and Houston (isolated in 2002 [EU310927]) GII.4 recombinant VLPs, which were kindly provided as a gift by Prof Mary Estes, Baylor College of Medicine, TX, USA.

### 2.3. RNA Extraction from Stool and Reverse Transcription

Viral RNA was extracted from stool samples using the QIAamp Viral RNA Mini Kit (Qiagen, Hilden, Germany) following the manufacturer’s protocol. Briefly, 140 µL of 10% stool suspension was lysed. After a series of washing steps, RNA was eluted with 60 µL AVE buffer. RNA from each sample was subsequently reverse transcribed using iScript™ Select cDNA Synthesis Kit (Bio-Rad, Uppsala, Sweden). Both gene-specific and random primers were initially evaluated. For the VP1 gene, random primers consistently yielded the best results (data not shown), and for the RdRp gene, a specific primer (UNP_135 at position 5271 in GII.4 genome) yielded the best results [36]. Briefly, 5 µL RNA was mixed with the reaction components to a final volume of 20 µL according to the manufacturer’s instructions. Reactions with random primers were incubated for 5 min at 25 °C, 60 min at 42 °C, and 5 min at 85 °C. Reactions with specific primers were incubated for 60 min at 42 °C and 5 min at 85 °C.

### 2.4. Sequencing of the Shell Region (for Genotyping) and the Entire VP1 Gene

Norovirus genotyping in 25 stool samples between 2013 and 2016 was performed by sequencing of the partial shellregion as described previously [37].

PCR amplification of the VP1 gene was conducted using iProof™ High-Fidelity PCR kit (Bio-Rad, Uppsala, Sweden) following the manufacturer’s protocol. Briefly, 15 µL of cDNA was mixed with the reaction components to a final volume of 75 µL. The final concentration of forward and reverse primer used was 0.15 µM. The primer sequences were obtained from literature or designed using NCBI primer design tool (Table 2). The reaction was carried out with the following cycling conditions: initial denaturation at 98 °C for 3 min, 35 cycles of denaturation at 98 °C for 10 s, annealing at 52 °C for 30 s, extension at 72 °C for 1.5 min, and a final extension at 72 °C for 7 min. If the quality of the first PCR amplicons (PCR1 in Table 2) was not satisfactory, another PCR using 2 primer pairs (PCR2a and 2b, Table 2) was conducted, with annealing temperature of 60 °C and extension time 1 min. The PCR products were purified through gel extraction using QIAquick Gel Extraction Kit (Qiagen, Hilden, Germany) following the manufacturer’s protocol. Purified amplicons were sent to Macrogen Inc. (Amsterdam, The Netherlands) for Sanger sequencing.

### 2.5. Sequencing of the RdRp Gene

PCR amplification of the RdRp gene was conducted using iProof™ High-Fidelity PCR kit (Bio-Rad, Uppsala, Sweden), following the manufacturer’s protocol. Briefly, 2.5 µL of cDNA was mixed with the reaction components to at final volume of 25 µL. Primers were obtained from literature or designed for this study, using NCBI primer design tool (Table 3). Different primer concentrations were evaluated: 0.15 µM, and in some cases 0.20 µM, yielded the best results and subsequently, these concentrations were used in the PCRs. The same cycling conditions were used as described for the PCR of the VP1 gene.

PCR products were visualized by electrophoresis on a 2% agarose gel, using SYBR™ Safe DNA Gel Stain and UV transillumination. PCR products suitable for sequencing were sent to Macrogen Inc (Amsterdam, The Netherlands) for Sanger sequencing.

If the quality of PCR products was not satisfactory, or the first PCRs did not yield the complete RdRp gene, new PCRs using new combinations of primer pairs (Table 3) and/or new cycling conditions, were conducted. Annealing time of 20 s and extension times of 60 s and 30 s were used for the shorter PCR amplicons.

### 2.6. Nucleotide Accession Numbers

Nucleotide sequences for the norovirus strains were deposited in Genbank with accession numbers VP1: ON116949-ON116958 and RdRp: ON116960-ON116971.

### 2.7. Data Preparation, Alignment, and Phylogenetics

Sanger sequencing chromatograms were manually examined. Double peaks with similar intensity were considered to originate from two quasispecies with a nucleotide variation at the specific position. Designated Xa or Xb (X referring to sample-ID), two sequences were constructed for each sample, where the double peaks were the only dissimilarities. No sequence had more than one double peak in a single codon. Reference sequences were obtained from GenBank (Supplementary Tables S1 and S2). All sequences were aligned by ClustalW multiple alignment in BioEdit (version 7.0.5.3). The best nucleotide substitution model for the two gene datasets (VP1 and RdRp) was selected based on the results of the Akaike Information Criterion (AIC) score calculated using MEGAX (version 10.2.6). All analysis was conducted on both Xa and Xb. Using the Maximum Likelihood (ML) algorithm in MEGAX, phylogenetic trees for VP1 and RdRp were constructed (with K2P substitution model with gamma distribution and K2P substitution model with gamma distribution with invariant sites, respectively). For simplicity, only Xa sequences are shown in the phylogenetic trees.

### 2.8. Analysis of the Protein Sequences of Immunodominant Epitopes and HBGA Binding Pockets of the VP1 Gene

Amino acid sequences of the VP1 protein of Den Haag variants (circulating 2006–2009) and patient sample sequences (collected 2013–2016) were aligned using ClustalW multiple alignment in BioEdit (version 7.0.5.3). The alignments were manually examined, specifically for determining amino acid substitutions in the proposed immunodominant epitopes and HBGA-binding pockets [7,15].

### 2.9. Analysis of the Protein Sequences of the RdRp Gene

Amino acid sequences of the RdRp protein in the patient samples were aligned by ClustalW multiple alignment in BioEdit (version 7.0.5.3). The alignment was manually examined to find if any amino acid substitutions occurred in the RdRp protein, during ribavirin treatment.

## 3. Results

### 3.1. The Patient Had Protective Blocking Titers in Serum

Saliva analysis showed that the patient was a secretor. Through administration of SCIg and IVIg (the patient had no intrinsic antibody production), one analyzed serum sample showed blocking titers comparable to immunocompetent individuals for both Grimsby (≥320) and Houston GII.4 VLPs (≥160. These titers are considered protective in immunocompetent individuals [33,34].

### 3.2. The VP1 and RdRp Genes Form Unique Subgroups in the GII.4 Den Haag 2006b Variant Cluster

**Figure 1:**
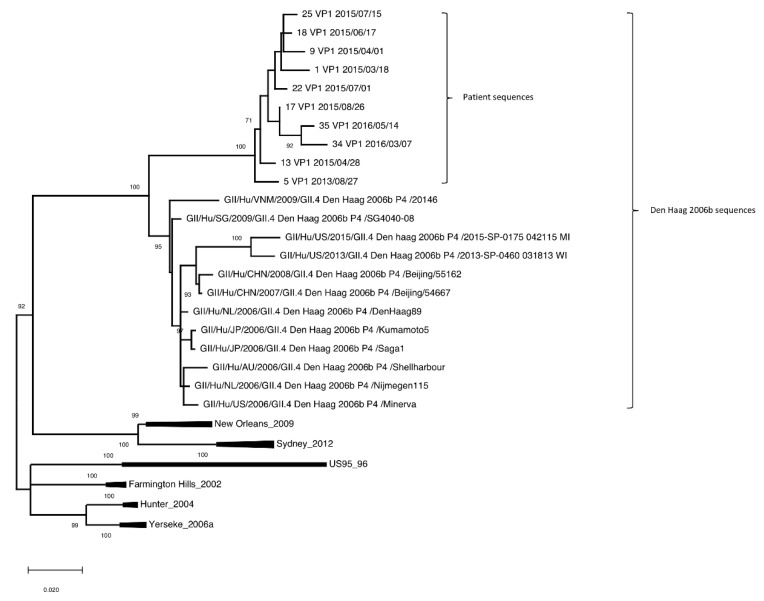
Phylogenetic tree built from VP1 gene of norovirus in the patient samples with reference sequences obtained from NCBI for comparison. Patient samples are designated with Sample-ID, gene name, and date of collection (YYYY/MM/DD). All Den Haag strains used for comparison are shown. Strains within other variants are minimized but can be found in Supplementary Table S1.

Noroviruses in 25 stool samples were genotyped as GII.4 following sequencing of the partial shell region of the VP1 gene and were highly similar to each other (≥99.4% nucleotide identity). Of these, the entire VP1 gene was successfully sequenced for 10 samples. Phylogenetic analysis showed that the norovirus sequences created a subcluster within the GII.4 Den Haag variant cluster and was most closely related to a Den Haag strain from 2009. All variants included in the tree (and the norovirus from the chronic infection), organized into distinct clusters separated with high bootstrap values (Figure 1). Obtained nucleotide sequences were compared to known norovirus sequences. The nucleotide sequences from the patient samples showed 94.2–95.1% identity with other GII.4 Den Haag variants circulating between 2006 and 2009 for the sample collected in 2013, 93.7–94.9% for samples collected in 2015, and 93.4–94.1% for samples collected in 2016.

**Figure 2:**
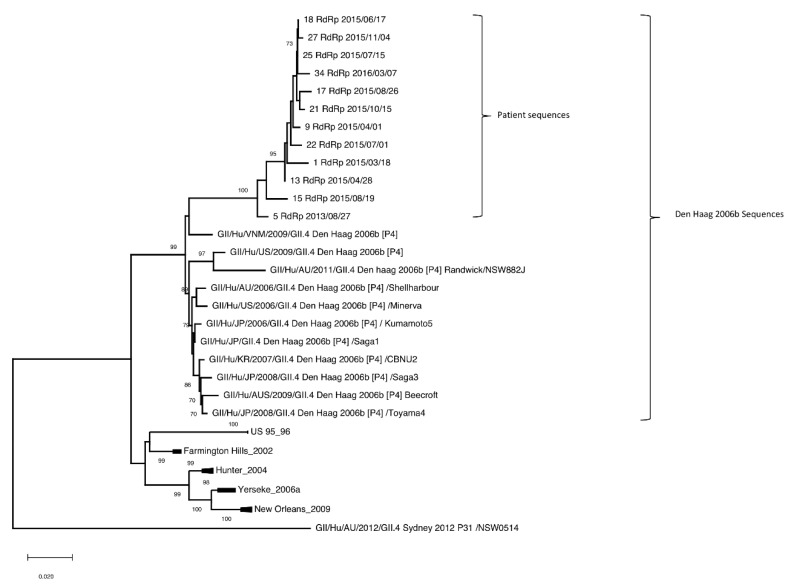
Phylogenetic tree built from RdRp gene of norovirus in the patient samples with reference sequences obtained from NCBI for comparison. Patient samples are designated with Sample-ID, gene name, and date of collection (YYYY/MM/DD). All Den Haag strains used for comparison are shown. Strains within other variants are minimized but can be found in Supplementary Table S2.

The RdRp gene was successfully sequenced in 12 samples. Phylogenetic analysis showed that the sequences form a subcluster within Den Haag GII.[P4] (P-type) variant cluster and also shared a most recent common ancestor (MRCA) with a Den Haag variant from 2009 (Figure 2). The obtained nucleotide sequences were compared to known norovirus sequences. The sequence from the sample collected in 2013 showed a genetic similarity of 96.1–96.5% compared to the closest known sequences, all GII.[P4] polymerases circulating between 2006 and 2009. Nucleotide identities for sequences from samples collected 2015 were 95.4–95.7% and for samples collected in 2016, were 95.1–95.4%.

### 3.3. Distinct Amino Acid Differences Were Found in Epitopes A, C, and D whereas HBGA Binding Epitopes Were Conserved

**Figure 3:**
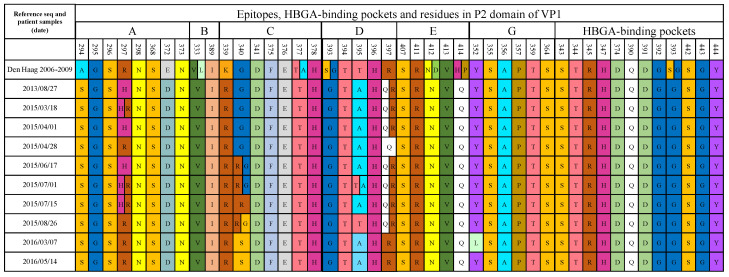
Amino acid changes within the proposed immunodominant epitopes of P2 domain of VP1 (A–E and G) [7] and HBGA binding pockets [15,16,17] among the patient sample sequences (date) and Den Haag 2006–2009 reference sequence for comparison.

Amino acid alignment of the VP1 gene was performed on the 10 patient sample sequences (2013–2016) as well as sequences of Den Haag 2006b variants circulating between 2006 and 2009. Roughly three years passed between the first and the last collected sample in both these groups. Within proposed immunodominant epitopes (A–E and G) and HBGA-binding pockets, differences in amino acid sequences between reference and patient samples were noted, as well as substitutions occurring during the chronic infection. Compared to references, patient samples exhibited distinct differences in several immunodominant epitopes. Substitutions occurred at residues 294, 297, and 372 in epitope A, at residues 339 and 340 in epitope C, at residues 395 and 397 in epitope D, and at residue 352 in epitope G in one sample. The residues of the HBGA binding pockets were completely conserved during the chronic infection (Figure 3).

Alignment with all reference GII.4 variants included in the phylogenetic trees (Figure 1 and Figure 2), demonstrated that VP1 of patient samples analyzed were 542 amino acids in length, compared to reference sequences which were 540 amino acids in length (except for the US 95_96 variant, which was 539 amino acids in length). This was attributable to an insertion of lysine and glutamic acid at residue 172–173, which could be observed in all 10 patient samples (data not shown).

### 3.4. Ribavirin Treatment Failed to Clear the Norovirus Infection

**Figure 4:**
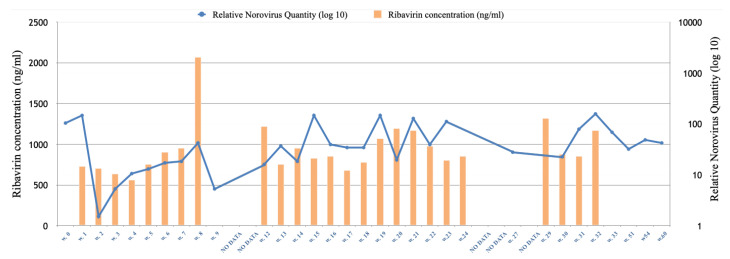
Serum analysis of ribavirin concentration (ng/mL in serum), designated in weeks (w) since started treatment. Treatment start = w. 0. Relative norovirus quantity is expressed as fold change compared to the screened sample with the highest qPCR Ct value (21.5). At some collection times, either fecal or serum sample could not be collected. NO DATA—no fecal sample and/or ribavirin concentration collected due to illness/treatment from other cause.

Blood ribavirin concentration compared to relative quantity of norovirus shedding in stool can be found in Figure 4. Ribavirin treatment failed to clear the norovirus infection despite a treatment duration of ~7.5 months. For most of the treatment span, the patient could not reach the target values of ribavirin >1000 ng/mL, due to side effects and other complications (Figure 4 and Table 1). No clear effect of ribavirin on relative norovirus quantity could be observed during the treatment. However, a decline was observed two weeks after treatment initiation, yielding the lowest quantity of norovirus shedding during treatment (Figure 4).

The RdRp gene was generally conserved during ribavirin treatment. Compared to the norovirus sample at treatment initiation, two amino acid substitutions at positions 370 (asparagine: >threonine) and 403 (valine: >phenylalanine) were observed two weeks after the first administered ribavirin dose and were subsequently preserved during treatment (Supplementary Figure S1). However, threonine and phenylalanine were also observed at positions 370 and 403, respectively, in an older norovirus sample collected in 2013 (81 weeks before treatment initiation).

## 4. Discussion

In this study, we describe a CVID patient with low immunoglobulin levels and late onset combined B- and T-cell deficiency, who contracted CVID-associated enteropathy coinciding with a chronic norovirus infection. The chronic norovirus infection was studied in the context of norovirus evolution, during a timespan in which an attempt to treat the infection with the antiviral ribavirin was made. Phylogenetic analysis of patient sample sequences showed distinct clusters of VP1 and RdRp genes, clustering together and creating unique subclusters in the GII.4 Den Haag 2006b [P4] variant cluster (Figure 1 and Figure 2). The unique subclusters were most closely related to Den Haag variants from 2009. It is therefore possible that the patient was infected by a norovirus Den Haag variant at some time around 2009, which also correlates to the medical history of the patient as symptoms of diarrhea first appeared around this time.

Through regularly administered SCIg and IVIg, one analyzed serum sample of the patient contained norovirus blocking titers comparable to levels associated with protection in immunocompetent individuals, but these were not sufficient to clear the infection. This is in line with earlier arguments that the humoral immune response with neutralizing antibodies alone may not be sufficient to clear norovirus infection, and that functioning T cell response also contribute [40]. The patient in this study had a form of CVID with impaired T lymphocyte function, possibly contributing to failure to clear the virus. However, it is important to note that blocking titers is a surrogate marker for protection and the patient also lacked IgA which could be a further contributing factor in failure to clear the infection. B cells have earlier been implicated as important for norovirus replication [41,42]. However, the norovirus infection persisted for years in this study even though B cells could not be detected in the patient. This finding supports previous observations that B cells are not necessary for productive norovirus infection [27,43,44,45].

Several amino acid substitutions were observed in epitopes A (294, 297, 372), C (339 and 340), and D (395 and 397) (Figure 3), when compared to other Den Haag 2006b reference sequences. During intra-host evolution, most of the substitutions occurred at residues 297, 340, and 397. Other studies of chronic norovirus infections have also observed multiple substitutions in epitopes A, C, and D, including residues 297 and 340 [15,46] and 397 [47]. Residue 297 in the immunodominant epitope A is suggested to have an important role in immune escape and is consistently altered between epidemic GII.4 strains. This residue has also been found to be under positive selection in chronic norovirus infections. Residue 340 is a variable residue of epitope C, which is frequently altered in epidemic strains and chronic infections [10,48]. It is located on the surface and lateral edge of the capsid and in direct proximity to the HBGA binding pocket. Residue 397 is part of a recently proposed extension of antigenic site D, whose specific significance warrants further study [7,10,15,48]. Several previous studies have used monoclonal antibody approaches to examine if changes in putative epitopes can help the virus to evade adaptive immune responses. Substitutions in site 340 led to alterations in monoclonal antibody binding capacity between wild-type and mutated VLPs [7,49]. Similar experiments and results are reported for residue 297 [50,51]. In contrast to the variation observed in antigenic sites A, C, and D, we observed a high degree of conservation in the proposed epitope G, suggested to play an important role in generating GII.4 strains with pandemic potential [7].

Together, these findings suggest that the norovirus in the chronically infected CVID patient was under some degree of selection pressure during the infection. This was possibly attributable to the SCIg and IVIg routinely given to the patient. However, it is also important to consider that this patient had a severely dysregulated immune response, with unknown effects on the selection pressure exerted on the norovirus. The effect from specific epitope changes on viral viability, infectivity, and/or pathogenicity is unknown. Several groups have further speculated whether chronic norovirus infections could act as reservoirs for the development of new strains of noroviruses with infective capabilities and outbreak-potential [33,34,42]. Results from this study and others show that novel and distinct GII.4 strains can emerge through intra-host evolution in immunocompromised hosts. The epidemiological importance of such chronic infections as a source of new strains is under debate [34,47,52,53]. To our knowledge, even though the patient had up to ~7 years of norovirus infection, no secondary cases were confirmed in family or healthcare personnel. However, no organized screening was carried out during that time. Moreover, although the sample size is small, there is no previous evidence of any GII.4 lineages having arisen and spread in the population from a chronically infected, immunocompromised host [52]. It is likely that norovirus, which is usually an acute infection, loses transmission capacity during long term adaptation to one host, as observed for many chronic infections [53]. The use of intestinal organoids in in vitro systems for norovirus study may shed more light on the viability and virulence of novel norovirus strains arising in a chronically infected host. Norovirus has been successfully replicated in intestinal organoids from chronically infected immunocompromised pediatric patients [54]. Another recent study attempted to replicate a GII.4 norovirus strain from a chronically infected CVID patient, but without success [24].

In this study, we can also report a unique 2 amino acid long insertion at residue 172–173 in the shell domain of VP1, which was observed in all sequenced patient samples. Despite being a presumably beneficial mutation for the virus, it is not known what effect this insertion could have in terms of viral viability, pathogenicity, and/or infectiousness in other hosts.

The residues of the HBGA-binding pockets were completely conserved between all sampling points and were highly similar to the Den Haag references sequences, in this secretor-positive host. This observation supports the importance of HBGA-binding in maintaining chronic infections, even in severely immunosuppressed individuals. Similar results have been reported from previous studies [46,55]. To our knowledge, there are no larger studies investigating the importance of secretor status in the development of chronic norovirus infection in immunocompromised patients, which should be addressed in future studies. Human milk oligosaccharides (HMOs), which structurally mimic HBGAs, have been shown to block norovirus VLPs from binding to HBGAs [55,56]. As such and given the conservation of the HBGA binding pocket throughout the chronic infection, HMOs may be a putative treatment candidate for chronic norovirus infections which warrants further study.

In a study by Woodward et al. [22,27], five CVID patients with norovirus-associated enteropathy were treated with oral ribavirin for up to 6 months. Viral clearance occurred in two patients following achievement of target serum levels of ribavirin >1000 ng/mL, which was associated with complete symptomatic and histological recovery. All patients were infected with separate strains of GII.4 genotypes of norovirus. In our study, however, ribavirin could not clear the chronic GII.4 norovirus infection. Target serum-levels of >1000 ng/mL could only be achieved in 7/22 of analyzed samples, which might be one reason for treatment failure. Unlike Woodward et al., and like others [24], we could not observe any clear association between ribavirin levels and quantity of norovirus shedding during the treatment period, possibly due to the generally low concentrations of ribavirin (Figure 4). However, a decline of norovirus concentration was observed two weeks after treatment initiation, which was followed by a gradual increase of viral concentrations over the following weeks. Interestingly, at the two-week time point, we observed two amino acid substitutions (on positions 370 and 403), which were maintained during treatment and after. However, these amino acid substitutions were also present in an older sample collected in 2013, making it hard to draw any conclusions on any putative association to ribavirin resistance. No other consistent amino acids substitutions in the RdRp gene were observed during treatment. A recent study observed an increase in nucleotide substitutions in the norovirus genome during ribavirin treatment, but the treatment did not clear the norovirus infection [24].

In summary, we report a chronic norovirus infection over several years in an immunocompromised patient with CVID. Oral ribavirin treatment and IVIg could not clear the infection. Evolutionary analysis over the course of ~3 years showed distinct changes in immunodominant epitopes in VP1, while HBGA binding sites were conserved. The observed evolutionary changes in the norovirus genome reveal the potential of chronic infections to generate novel norovirus strains; however, the epidemiological importance of this remains elusive.

## Figures and Tables

**Table 1:** Date of sample collection (fecal and/or serum), weeks since start of ribavirin-treatment (minus-symbol used to designate before initiation of treatment), ribavirin concentration in serum, ID-code of faecal sample and if any of VP1 or RdRp gene or both were sequenced successfully. The target concentration of ribavirin was 1000 ng/mL. No sample collected: sample collection was not possible. NA: sample collected and analyzed for norovirus by qPCR but not available for sequencing.

Date of Sample Collection	Week Since Initiated Ribavirin Treatment	Ribavirin Concentration (ng/mL)	ID-Code of Faecal Sample	Sequenced Genes
2013/08/27	−81		5	VP1 and RdRp
2015/03/18	0	start ribavirin	1	VP1 and RdRp
2015/03/25	1	732	NA	none
2015/04/01	2	707	9	VP1 and RdRp
2015/04/07	3	634	10	none
2015/04/15	4	561	11	none
2015/04/22	5	756	12	none
2015/04/28	6	902	13	VP1 and RdRp
2015/05/05	7	951	14	none
2015/05/13	8	2073	30	none
2015/05/20	9	no sample collected	31	none
-	10	no sample collected	no sample collected	none
-	11	no sample collected	no sample collected	none
2015/06/10	12	1220	16	none
2015/06/17	13	756	18	VP1 and RdRp
2015/06/24	14	951	20	none
2015/07/01	15	829	22	VP1 and RdRp
2015/07/08	16	853	24	none
2015/07/15	17	683	25	VP1 and RdRp
2015/07/22	18	780	NA	none
2015/07/29	19	1073	NA	none
2015/08/05	20	1195	28	none
2015/08/12	21	1171	29	none
2015/08/19	22	976	15	RdRp
2015/08/26	23	805	17	VP1 and RdRp
2015/09/02	24	854	no sample collected	none
-	25	no sample collected	no sample collected	none
-	26	no sample collected	no sample collected	none
2015/09/23	27	no sample collected	19	none
-	28	no sample collected	no sample collected	none
2015/10/06	29	1317	no sample collected	none
2015/10/15	30	878	21	RdRp
2015/10/21	31	854	23	none
2015/10/28	32	1171	26	none
2015/11/04	33	End ribavirin	27	RdRp
2016/03/07	51		34	VP1 and RdRp
2016/04/01	54		32	none
2016/05/14	60		35	VP1

**Table 2:** Primer sequences used for PCR reactions and Sanger sequencing of the complete VP1 gene. Positions mapped in GII.4 genome (accession number KM198570). Tm indicates the annealing temperature used in the present study.

Primer	Used in	Sequence (5′ -> 3′)	Tm (°C)	Position	References
VP1_F1	PCR 1 + Sequencing	CAAGAGCCAATGTTCAGATGG	52	4982–5003	[38]
VP1_F2	PCR 2a + Sequencing	GAGTGACGCCAACCCATCTAA	60	5067–5088	This study
VP1_F3	PCR 2b + Sequencing	CCACCCACAGTTGAGTCAAGA	60	5717–5738	This study
VP1_R1	PCR 1 + Sequencing	GACATCAGATGCCAATCCAG	52	6726–6746	This study
VP1_R2	PCR 2a + Sequencing	TGACTCAACTGTGGGTGGCA	60	5755–5795	This study
VP1_R3	PCR 2b + Sequencing	ATAAAGCACGTCTACGCCCC	60	6710–6730	This study
VP1_F4	Sequencing	CACCACTTAGGGCYAAYAATGCTGG	52	5635–5659	[39]
VP1_F5	Sequencing	GATGTCACCCACATTGCAGGTTCTCG	52	5949–5974	[39]
VP1_R4	Sequencing	CCAGCATTRTTRGCCCTAAGTGGTG	52	5635–5659	[39]
VP1_R5	Sequencing	CGAGAACCTGCAATGTGGGTGACATC	52	5949–5974	[39]

**Table 3:** Primer sequences used for PCR reactions and Sanger sequencing of the complete RdRp gene. Primer position mapped in Norovirus GII.4 genome (accession nr. EF684915.2). Tm indicates the annealing temperature used in the present study.

Primer	Used in	Sequence (5′ -> 3′)	Tm (°C)	Position	References
RDRP_F1	PCR and sequencing	TGYCCCTAYATCTACAAGAG	52	3449–3468	This study
RDRP_F2	PCR and sequencing	ACACAGCTGCACTYAAGGAT	52	4054–4073	This study
RDRP_F3	PCR and sequencing	AAGCTCAAGGAGTATGGGTTG	52	4652–4672	This study
RDRP_F4	PCR and sequencing	TGGCAGCTGCTCTAGAAATCATG	52	4335–4357	This study
UNP_135	PCR and sequencing	GACCTCTGGGACGAGGTTG	52	5132–5150	[36]
RDRP_R2	Sequencing	TCTGATCCAATTTTCCAAAC	52	4777–4796	This study
RDRP_R3	PCR and sequencing	CAGTGTGCTTTGAGTTCATC	52	4181–4200	This study
RDRP_R4	PCR and sequencing	GGAAGACCCTCGTTGATTG	52	4449–4467	This study
RDRP_R5	PCR and sequencing	GTTGGTTTCAACCCATACTC	52	4661–4680	This study
RDRP_R6	PCR and sequencing	CAGTTCTCCGCAGGAAAGTC	52	4735–4754	This study

## Data Availability

The data in the present study are available upon request to the authors.

## Supplementary Materials

The following supporting information can be downloaded at: https://www.mdpi.com/article/10.3390/v14081708/s1, Figure S1: Amino acid alignment of a segment of the RdRp gene; Table S1: Name and accession number of reference sequences obtained from GenBank used to create the phylogenetic tree and alignment comparison for VP1 gene; Table S2: Name and accession number of reference sequences obtained from GenBank used to create the phylogenetic tree and alignment comparison for RdRp gene.
