# Supplementary material for: Long Term Norovirus Infection in a Patient with Severe Common Variable Immunodeficiency
Source: Viruses. 2022 Aug 2;14(8):1708. doi: 10.3390/v14081708 (PMC9413339; doi:10.3390/v14081708)
Supplement: Supplementary file 1 [file viruses-14-01708-s001.zip › viruses-1798877-supplementary.pdf]

Table S1. Name and accession number of reference sequences obtained from GenBank used to create the phylogenetic tree and alignment comparison for VP1 gene.

Strain	Accession number
GII/Hu/AU/1994/GII.4 US95_96 [P4]/Camberwell	U46500
GII/Hu/UK/1995/GII.4 US_95_96 [P4]	AJ004864
GII/Hu/US/2002/GII.4 Farmington Hills_2002 [P4]/Farmington Hills_2002	AY502023
GII/Hu/DE/2002/GII.4 Farmington Hills [P4] /Langen1061	AY485642
GII/Hu/US/2004/GII.4 Farmington_Hills_2002 [P4]/Farmington Hills	JQ478408
GII/Hu/TUN/2002/GII.4 Hunter_2004 [P4]/11453	KU182482
GII/Hu/NL/2004/GII.4 Hunter_2004 [P4]/0317	AY883096
GII/Hu/CHN/2005/GII.4 Hunter_2004 [P4]/CU050140	HM802542
GII/Hu/US/2006/GII.4 Den Haag_2006b [P4]/Minerva	KT152148
GII/Hu/AU/2006/GII.4 Den Haag_2006b [P4]/Shellharbour	EF684915
GII/Hu/JP/2006/GII.4 Den Haag_2006b [P4]/Saga1	AB447456
GII/Hu/NL/2006/GII.4 Den Haag_2006b [P4]/Nijmegen115	EF126966
GII/Hu/NL/2006/GII.4 Den Haag_2006b [P4]/DenHaag89	EF126965
GII/Hu/JP/2006/GII.4 Den Haag_2006b [P4]/Kumamoto5	AB447463
GII/Hu/CHN/2007/GII.4 Den Haag_2006b [P4]/Beijing/54667	GQ856461
GII/Hu/CHN/2008/GII.4 Den Haag_2006b [P4]/Beijing/55162	GQ856458
GII/Hu/SG/2009/GII.4 Den Haag_2006b [P4]/SG4040-08	JX459641
GII/Hu/VNM/2009/GII.4 Den Haag_2006b [P4]/20146 GII/Hu/US/2013/GII.4 Den Haag_2006b	KM198570
[P4]/2013-SP-0460_031813_WI	KX354091
GII/Hu/US/2015/GII.4 Den_haag_2006b [P4]/2015-SP-0175_042115_MI	KX354128
GII/Hu/AU/2008/GII.4 Yerseke_2006a [P4]/NSW023C	GQ849126
GII/Hu/NL/2006/GII.4 Yerseke_2006a [P4]/Terneuzen70 GII/Hu/NL/2006/GII.4 Yerseke_2006a	EF126964
[P4]/Yerseke38	EF126963
GII/Hu/TW/2006/GII.4 Yerseke_2006a [P4]	KM245069
GII/Hu/US/2008/GII.4 New Orleans_2008 [P4]/New Orleans1500	GU270580
GII/Hu/US/2009/GII.4 New Orleans_2009 [P4]/New Orleans1805	GU445325
GII/Hu/US/2010/GII.4 New Orleans_2009 [P4]	JN595867
GII/Hu/TW/2010/GII.4 New Orleans_2009 [P4]/CGMH25	JN400623
GII/Hu/AU/2012/GII.4 Sydney_2012 [P31]/Woonona	JX459907
GII/Hu/AU/2012/GII.4 Sydney_2012 [P31]/NSW0514	JX459908
GII/Hu/JP/2016/GII.4 Sydney_2012 [P16]/OH16002	LC153121
GII/Hu/JP/2016/GII.4 Sydney_2012[P16]/Kawasaki194	LC175468

Table S2. Name and accession number of reference sequences obtained from GenBank used to create the phylogenetic tree and alignment comparison for RdRp gene:.

Strain	Accession number
GII/Hu/US/2001/GII.4 US95_96 [P4]/HS66	KJ407076
GII/Hu/US/2004/GII.4 US95_96 [P4]/HS191	KC013592
GII/Hu/US/2002/GII.4Farmington_Hills_2002 [P4]/Farmington Hills	AY502023
GII/Hu/UK/2002/GII.4 Farmington_Hills_2002 [P4]/Farmington Hills	AY587983
GII/Hu/CHN/2005/GII.4 Hunter_2004[P4]/Hong Kong	HM802542
GII/Hu/US/2008/GII.4 Hunter _2004 [P4]	MH413070
GII/Hu/AU/2005/GII.4 Hunter_2004[P4]/Hunter504D/04O	DQ078814
GII/Hu/AU/2006/GII.4 Den Haag_2006b [P4] /Shellharbour	EF684915
GII/Hu/JP/2006/GII.4 Den Haag_2006b [P4]/ Kumamoto5	AB447463
GII/Hu/US/2009/GII.4 Den Haag_2006b [P4]	GU325839
GII/Hu/US/2006/GII.4 Den Haag_2006b [P4]/Minerva	KT152148
GII/Hu/JP/GII.4 Den Haag_2006b [P4]/Saga1	AB447456

GII/Hu/VNM/2009/GII.4 Den Haag_2006b [P4]	KM198570
GII/Hu/AU/2009/GII.4 Den Haag_2006b [P4] Beecroft	HM748971
GII/Hu/AU/2011/GII.4 Den haag_2006b [P4]Randwick/NSW882J	JX459900
GII/Hu/JP/2008/GII.4 Den Haag_2006b [P4]/Toyama4	AB541360
GII/Hu/JP/2006/GII.4 Yerseke_2006a [P4]/Aomori1	AB447432
GII/Hu/NL/2012/GII.4 New Orleans_2009 [P4]/Nijmegen01	LN854571
GII/Hu/US/2009/GII.4 New Orleans_2009 [P4]/New Orleans1805	GU445325
GII/Hu/US/2010/GII.4 New Orleans_2009 [P4]	JN595867
GII/Hu/AU/2012/GII.4 Sydney_2012 [P31]/NSW0514	JX459908
GII/Hu/KR/2007/GII.4 Den Haag_2006b [P4]/CBNU2	JQ622197
GII/Hu/JP/2008/GII.4 Den Haag_2006b[P4]/Saga3	AB541334
GII/Hu/US/2011/GII.4 Yerseke_2006a [P4]/MI001	KC631814

Figure S1. Amino acid alignment of a segment of the RdRp gene. Patient samples are designated with weeks (W) since initiation of ribavirin treatment and sample-ID (in parenthesis). (+) indicates weeks after end of ribavirin treatment. (−) indicates weeks before initiation of ribavirin treatment.

RdRp gene alignment (residue 364-408)

	364	365	366	367	368	369	370	371	372	373	374	375	376	377	378	379	380	381	382	383	384	385	386	387	388	389	390	391	392	393	394	395	396	397	398	399	400	401	402	403	404	405	4-06	407	408
RdRp W 0 (1)	E	Y	G	L	K	P	N	R	P	D	K	T	E	G	P	L	V	I	S	E	D	L	N	G	L	T	F	L	R	R	T	V	T	R	D	P	A	G	W	V	G	K	L	D	Q
RdRp W 2 (9)	-	-	-	-	-	-	T	-	-	-	-	-	-	-	-	-	-	-	-	-	-	-	-	-	-	-	-	-	-	-	-	-	-	-	-	-	-	-	-	F	-	-	-	-	-
RdRp W 6 (13)	-	-	-	-	-	-	T	-	-	-	-	-	-	-	-	-	-	-	-	-	-	-	-	-	-	-	-	-	-	-	-	-	-	-	-	-	-	-	-	F	-	-	-	-	-
RdRp W 13 (18)	-	-	-	-	-	-	T	-	-	-	-	-	-	-	-	-	-	-	-	-	-	-	-	-	-	-	-	-	-	-	-	-	-	-	-	-	-	-	-	F	-	-	-	-	-
RdRp W 15 (22)	-	-	-	-	-	-	T	-	-	-	-	-	-	-	-	-	-	-	-	-	-	-	-	-	-	-	-	-	-	-	-	-	-	-	-	-	-	-	-	F	-	-	-	-	-
RdRp W 17 (25)	-	-	-	-	-	-	T	-	-	-	-	-	-	-	-	-	-	-	-	-	-	-	-	-	-	-	-	-	-	-	-	-	-	-	-	-	-	-	-	F	-	-	-	-	-
RdRp W 22 (15)	-	-	-	-	-	-	T	-	-	-	-	-	-	-	-	-	-	-	-	-	-	-	-	-	-	-	-	-	-	-	-	-	-	-	-	-	-	-	-	F	-	-	-	-	-
RdRp W 23 (17)	-	-	-	-	-	-	T	-	-	-	-	-	-	-	-	-	-	-	-	-	-	-	-	-	-	-	-	-	-	-	-	-	-	-	-	-	-	-	-	F	-	-	-	-	-
RdRp W 30 (21)	-	-	-	-	-	-	T	-	-	-	-	-	-	-	-	-	-	-	-	-	-	-	-	-	-	-	-	-	-	-	-	-	-	-	-	-	-	-	-	F	-	-	-	-	-
RdRp W 33 (27)	-	-	-	-	-	-	T	-	-	-	-	-	-	-	-	-	-	-	-	-	-	-	-	-	-	-	-	-	-	-	-	-	-	-	-	-	-	-	-	F	-	-	-	-	-
RdRp W +18 (34)	-	-	-	-	-	-	T	-	-	-	-	-	-	-	-	-	-	-	-	-	-	-	-	-	-	-	-	-	-	-	-	-	-	-	-	-	-	-	-	F	-	-	-	-	-
RdRp W -81 (5)	-	-	-	-	-	-	T	-	-	-	-	-	-	-	-	-	-	-	-	-	-	-	D	-	-	-	-	-	-	-	-	-	-	-	-	-	-	-	-	F	-	-	-	-	-
